# A spatial individual-based model predicting a great impact of copious sugar sources and resting sites on survival of *Anopheles gambiae* and malaria parasite transmission

**DOI:** 10.1186/s12936-015-0555-0

**Published:** 2015-02-05

**Authors:** Lin Zhu, Whitney A Qualls, John M Marshall, Kris L Arheart, Donald L DeAngelis, John W McManus, Sekou F Traore, Seydou Doumbia, Yosef Schlein, Günter C Müller, John C Beier

**Affiliations:** Department of Public Health Sciences, Miller School of Medicine, University of Miami, Miami, Florida USA; Department of Infectious Disease Epidemiology, MRC Centre for Outbreak Analysis and Modelling, Imperial College London, London, UK; USGS/Biological Resources Division and Department of Biology, University of Miami, Coral Gables, Florida USA; Department of Marine Biology and Ecology, University of Miami, Miami, Florida USA; Malaria Research and Training Center, Faculty of Medicine, Pharmacy and Odonto-Stomatology, University of Bamako, BP 1805 Bamako, Mali; Department of Microbiology and Molecular Genetics, IMRIC, Kuvin Centre for the Study of Infectious and Tropical Diseases, Faculty of Medicine, Hebrew University, Jerusalem, Israel

**Keywords:** Malaria, *Anopheles gambiae*, Sugar-feeding, Resting, Behavior, Individual-based model, Agent-based model

## Abstract

**Background:**

Agent-based modelling (ABM) has been used to simulate mosquito life cycles and to evaluate vector control applications. However, most models lack sugar-feeding and resting behaviours or are based on mathematical equations lacking individual level randomness and spatial components of mosquito life. Here, a spatial individual-based model (IBM) incorporating sugar-feeding and resting behaviours of the malaria vector *Anopheles gambiae* was developed to estimate the impact of environmental sugar sources and resting sites on survival and biting behaviour.

**Methods:**

A spatial IBM containing *An. gambiae* mosquitoes and humans, as well as the village environment of houses, sugar sources, resting sites and larval habitat sites was developed. *Anopheles gambiae* behaviour rules were attributed at each step of the IBM: resting, host seeking, sugar feeding and breeding. Each step represented one second of time, and each simulation was set to run for 60 days and repeated 50 times. Scenarios of different densities and spatial distributions of sugar sources and outdoor resting sites were simulated and compared.

**Results:**

When the number of natural sugar sources was increased from 0 to 100 while the number of resting sites was held constant, mean daily survival rate increased from 2.5% to 85.1% for males and from 2.5% to 94.5% for females, mean human biting rate increased from 0 to 0.94 bites per human per day, and mean daily abundance increased from 1 to 477 for males and from 1 to 1,428 for females. When the number of outdoor resting sites was increased from 0 to 50 while the number of sugar sources was held constant, mean daily survival rate increased from 77.3% to 84.3% for males and from 86.7% to 93.9% for females, mean human biting rate increased from 0 to 0.52 bites per human per day, and mean daily abundance increased from 62 to 349 for males and from 257 to 1120 for females. All increases were significant (P < 0.01). Survival was greater when sugar sources were randomly distributed in the whole village compared to clustering around outdoor resting sites or houses.

**Conclusions:**

Increases in densities of sugar sources or outdoor resting sites significantly increase the survival and human biting rates of *An. gambiae* mosquitoes. Survival of *An. gambiae* is more supported by random distribution of sugar sources than clustering of sugar sources around resting sites or houses. Density and spatial distribution of natural sugar sources and outdoor resting sites modulate vector populations and human biting rates, and thus malaria parasite transmission.

## Background

Malaria parasites are transmitted through the bites of anopheline mosquitoes, and the intensity of transmission largely depends on survival and human biting behaviour of the vector [1,2]. To survive, both male and female mosquitoes feed on sugar for energy [3]. Male mosquitoes depend exclusively on sugars for nutrients, while female mosquitoes feed on sugars for daily energy requirements, (e.g., flying, mating, etc.), and feed on blood for egg development, although blood could also be used as energy when sugar sources are completely unavailable [4]. Females need sugar which they require soon after emergence and sometimes also before blood feeding [3,5]. Optimum sugar feeding can prolong the mosquito lifespan and fecundity [6]. Population size and daily survival rates of anopheline mosquitoes are higher in natural sugar rich areas than in natural sugar poor areas [7,8]. Sugar source availability can also affect the sequence of behaviours after emergence [9,10]. A laboratory experiment showed that female *Anopheles gambiae* feed on sugar significantly more frequently in the absence of a blood source [11]. Conversely, as sugar and blood are energetically interchangeable, blood feeding frequency can increase when sugar sources are not available [5,8,11,12], and disturb the timing for oviposition and gonotrophic cycle [5]. Accordingly, the reduction of sugar sources can reduce the survival and the abundance of anopheline mosquitoes, but increase blood seeking and feeding frequency of each female mosquito at the same time; therefore, the impact of natural sugar sources on malaria transmission remains unclear.

A major part of adult mosquito life is spent in resting sites [13,14]. While different species have various diel activity patterns, anopheline mosquitoes mainly rest during the daytime and complete activities like sugar feeding and blood feeding during the night, and then return to resting sites [13]. Difficulty in finding a suitable resting site can result in additional flight time, which consumes more energy, leading to increased demand of sugar intake. Thus, availability of resting sites may affect their sugar-feeding behaviour and survival. In addition, if mosquitoes cannot find proper resting sites, they have a higher likelihood of being exposed to heat and sunlight, thereby increasing dehydration and mortality rate [15].

Readily available sugar sources and resting sites promote the survival and biting behaviour of anopheline mosquitoes, and as a byproduct they also affect their ability to transmit malaria parasites. However, there is limited research that addresses sugar-feeding and resting behaviours compared to blood feeding behaviour. The reason could be that sugar-feeding and resting behaviours are not directly associated with malaria transmission, which provokes less interest and limited research funding [16]. In addition, tracking sugar-feeding and resting behaviours of mosquitoes in field studies can be very difficult and have problems such as ethical issues [16]. With information from studies on the sugar-feeding and resting habits of malaria vectors, agent-based modelling (ABM)/individual-based modelling (IBM) can be an exceptionally suitable tool for predicting the outcome of given situations on anopheline survival by including these factors and simulating the interactions between the mosquitoes and their environment of sugar sources, resting sites, larval habitat sites, houses, and humans.

Several ABMs have been developed to simulate mosquito life cycles and their interactions with humans, and to evaluate vector control applications of larval source management (LSM), long-lasting insecticidal nets (LLINs) and indoor residual spraying (IRS) [17-21]. However, only few modelling studies included sugar feeding and resting behaviours. One previous mathematical modelling study incorporated sugar feeding of mosquitoes and estimated the daily sugar feeding rates at a field site in Mali [22]. It also examined the potential effectiveness of combining attractive toxic sugar baits (ATSB) [23] targeting the sugar-feeding behaviour with other vector control methods, such as LLINs and IRS [22]. However, this and other models lack spatial components (locations of objects, distances between objects, etc.), which limit the ability to estimate the impact of spatial configuration of objects in the environment. Another study developed a dynamic state variable model to predict the decisions of female mosquitoes selecting different behaviours including sugar-feeding, blood-feeding and ovipositing based on the physiological state and location of the mosquito. However, it emphasized the behaviour decisions and the location was only identified as indoors or outdoors [24]. In addition, current IBMs have simulated the interactions with time-step resolutions at hourly or even daily intervals [20], assuming that mosquitoes remain in the same state and perform only one behaviour in a whole hour or day, which is not realistic. Also, in current spatial IBMs, landscapes were set to be composed of a course grid of a small number of grid cells (e.g. 40 grid cells [17]), so each grid cell represents a large area. During each step mosquitoes would move a whole grid cell, reducing the amount of realistic stochasticity in the mosquito movement. The host seeking functions in the models also assumed that the mosquitoes could always find the resources in the eight adjacent grids, no matter what the target was and how much area one grid cell represented [17,20]. However, the attractiveness or attractive ranges of different resources/targets can be very different [25-27]. Hence, the success rate of resource-seeking, including human host-seeking, in current IBMs can be biased, leading to inaccurate estimation of human biting and malaria transmission. Further, human hosts in current IBMs are always static (representing humans sleeping in houses) in assigned grids [17,20]. However, with the use of vector control LLINs and IRS, indoor biting has been demonstrated to shift from indoors to outdoors [28,29]. Thus, if human hosts remain static indoors, there can be a decrease in host availability to mosquitoes that are far from house locations; this obviously leads to an underestimation of outdoor human biting rates, and also leads to female mosquitoes more concentrated around houses.

To estimate the impact of environmental sugar sources and outdoor resting sites on the survival and human biting rate of *An. gambiae*, a spatial IBM was developed that takes into consideration the sugar-feeding and resting behaviours, and aforementioned simplifications assumed in other models. The IBM used a continuous landscape, a time-step resolution of one second, specific attractive distances of different objects, moving human hosts, and *An. gambiae* with functions of sugar-feeding and resting behaviours. This model is the first IBM to examine both sugar-feeding and resting behaviours in the mosquito life cycle, and consider the potential impact of environmental structure of sugar sources and resting sites on malaria transmission. This study provides a basis for evaluating new vector control interventions targeting sugar feeding or resting behaviours.

## Methods

The ODD (Overview, Design concepts, and Details) protocol was developed in 2006 [30] and revised in 2010 [31] to standardize the description of ABM/IBM in publications. It helps to make the ABM/IBM more understandable, complete, and reproducible. The ODD protocol [31] is used here to describe the model.

### Purpose

The purpose of the model is to estimate the impact of the density and configuration of environmental sugar sources and outdoor resting sites on the survival and human biting rate of *An. gambiae* in a village setting.

## Entities, state variables, and scales

### Entities

#### Living entities

The living entities consisted of two types of agents: humans and *An. gambiae* mosquitoes.

##### Humans

A total of 60 humans were randomly assigned to the 20 houses in the center of the village and all assignments remained stable during the course of each repetition for the simulations.

##### Mosquitoes

The population of *An. gambiae* mosquitoes, males and females individually, were simulated over their individual lifetimes. The number of living mosquitoes could change depending on birth and death rates through time.

#### Non-living entities

The non-living entities consisted of a village landscape, houses, outdoor resting sites, sugar sources and larval habitat sites.

##### Landscape

A continuous two-dimensional space with a reflecting boundary was used to simulate the landscape. This allowed the moving agents (mosquitoes or humans) that would hit one of the boundaries to be reflected back instead of being permanently removed (absorbing boundary) or entering from the opposite side (non-absorbing boundary). Reflecting boundaries are considered more realistic for this study because *Anopheles* mosquitoes do not usually fly too far from their breeding habitats [32], and people in the village who may reach/move out of the village boundary are more likely to return from the same boundary.

##### Village and houses

Because the maximum flight distance of *An. gambiae* is estimated to be 200 to 400 metres [33], also in accordance with the common size of a village in Mali, the area of the village in the IBM was set to be 600 × 600 metres. In accordance with geographic configuration in Mali, the 20 houses were set to be randomly located in the center of the village in a grid of 100 × 100 metres. Once the location of each house was determined, it remained the same through all the simulation scenarios and repetitions.

##### Outdoor resting sites

A number of natural outdoor resting sites were used, and the locations were randomly selected and constant throughout all repetitions of each simulation, though densities of resting sites could differ between simulations. Houses were also considered to be resting sites but not counted in the number of outdoor resting sites.

##### Sugar sources

A number of sugar sources were used and scattered randomly throughout the area in simulation scenarios of different sugar source densities and scenarios of random distribution of sugar sources. In other scenarios, distributions adjacent to outdoor resting sites or houses, sugar sources were placed at the same locations of resting sites or houses. The numbers and locations remain constant throughout all repetitions of each simulation, but could change from simulation to simulation.

##### Larval habitat sites

Fifty larval habitat sites were scattered randomly throughout the area and remained constant throughout all simulations.

#### State variables

Each mosquito was characterized by variables for age (number of time steps since emergence), sugar (numbers representing the extent a mosquito needs sugar meals), blood (in females), and gravid status (in females, numbers representing pre-gravid, and length in days being gravid), as well as its locations (coordinates) in space: at a resting site, at a sugar source, at a larval habitat site (in females), on a human (in females), or in targeted or random movement. These variables represent their states/status in each step. Each human was characterized by variables for bites and their location in space. Each larval habitat site was characterized by variables for eggs (number of eggs oviposited in each aquatic site per day) and its location in space. All other agents have variables of locations in space.

#### Temporal scales

The time-step resolution was one second, meaning that each step represented one second. Simulations were performed over periods of 60 days.

### Process overview and scheduling

#### Humans

Once homes were assigned to humans, they remained the same through each repetition of simulations. The humans had functions of random movement and targeted movement: beginning at 07:00 each day, they moved out of their homes in random directions at each step; beginning at 20:00, they moved back, targeting their assigned homes until they arrived; then they remained at home during the night. When a human got a bite from a female *An. gambiae*, the bite counter would increase by 1.

#### Anopheles gambiae

**Figure 1:**
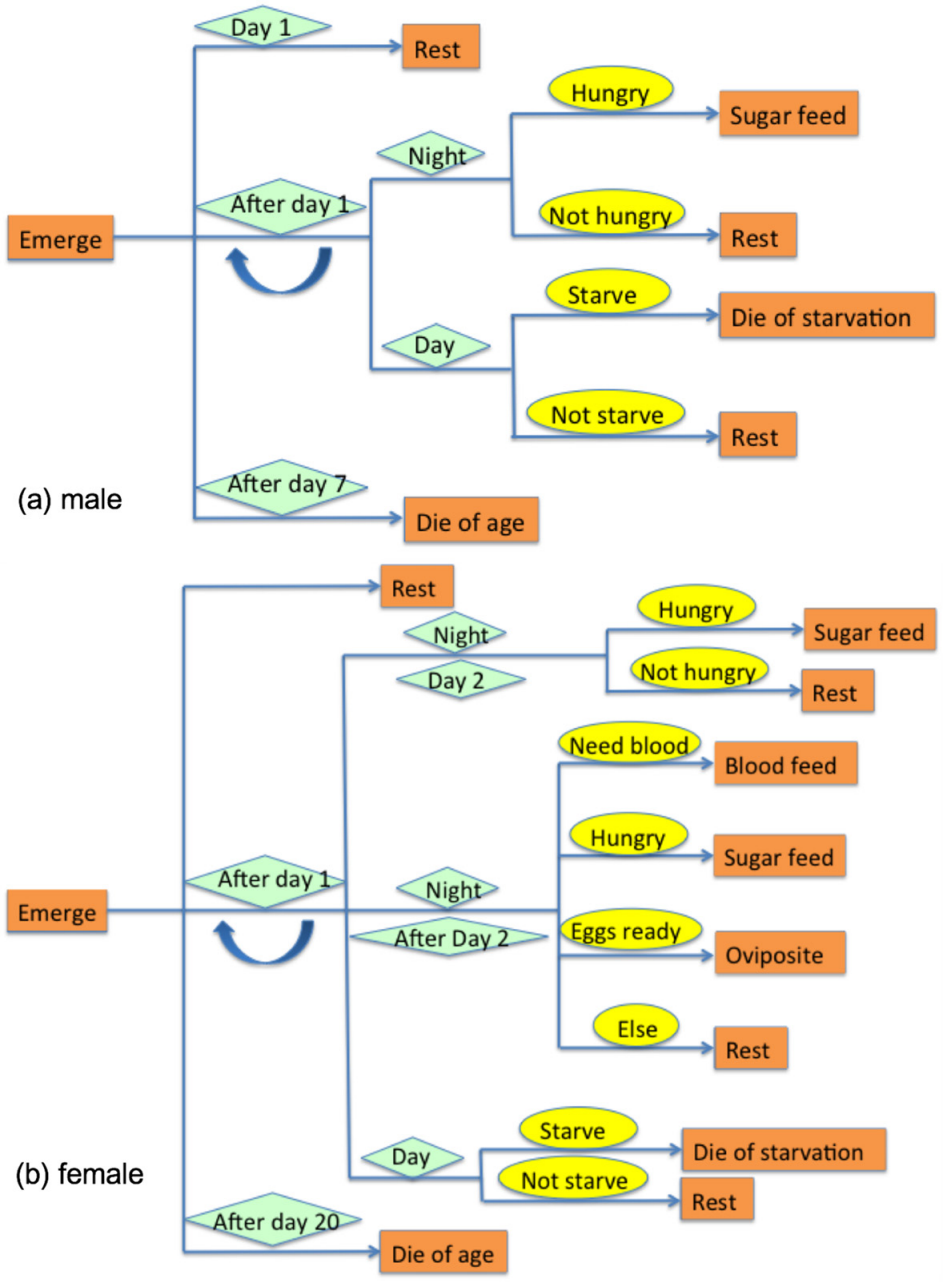
**Life cycle of** ***An. gambiae.*** Subfigures **(a)** and **(b)** represent life cycles of male *An. gambiae* and female *An. gambiae*. Orange rectangles represent the behaviours of the individuals, green diamonds represent the timing conditions, and the yellow ovals represent the status of the individuals. In each step, each *An. gambiae* would check its status and select a route in the figure.

The life cycles of an adult *An. gambiae* simulated in the model is shown in Figure 1. An average of 7 days was used for the longest life span for males [34]. From day 2, each individual male *An. gambiae* looped between behaviours until mortality was recorded. An estimate of 21 days was used for the longest life span for females [34]. Successful mating of every female was assumed. For females needing a blood meal, if it was not available during a 5 hour time span at night, the mosquito would switch to sugar-seeking [35]. Variable “sugar” records changes in need of sugar meal, and triggers sugar source seeking. Males needed at least two sugar meals per night while females did not have a minimal sugar meal requirement if they could get blood meals [36]. However, for both males and females, every additional flight of 2,000 steps, which generally represented 2000 metres, was assumed to lead to an additional need of one sugar meal [4]. At the beginning of each day, variable “sugar” of every mosquito will increase by one (need for one sugar meal) to count for energy consumption while resting. Mosquitoes that were not able to find an energy resource for a whole night would die of starvation. Blood-fed females would need two to three days to be ready to oviposit [37]. The number of eggs produced by gravid female *An. gambiae* was 100, variations due to individual physiological fitness and different blood meal size were not considered [38]. The development of their aquatic stages is described in the recruitment submodel section below.

#### Sequence of actions

The actions of each agent were considered sequentially. The agent being considered is called the ‘active’ agent, while any agent that is being acted on by the active agent is called the ‘passive’ agent. In each step, each active agent began by checking its own status, and then the desired movement type was decided based on the status of the agent. After that, the active agents would check the surrounding environment, and make spatial movement based on the environment and movement rules described earlier in the *An. gambiae* and human section. Finally the state variables of the agent would be updated as the result of the movement. If an interaction with another agent (usually a passive agent) occurred, a signal would also be sent to change the status of the other agent. The passive agents, on the other hand, would receive the signal from the interaction and change its state variable values accordingly. For example, in one step, age, sugar, blood, and gravid status of a female *An. gambiae* would be checked by the model, as well as the time of day; then, using that information, the model would decide if that female would seek a sugar source/blood source/larval habitat site/resting site or stay static. If the female mosquito needed a blood meal, then she would search the surrounding environment (usually a circle with a radius of the attractive distance of human) and see if there was a human within range. If there was then the female mosquito would make a targeted movement of one meter toward the human. With its movement type variable marked as targeted, she would continue to fly toward the human in the following steps without checking her status to decide desired movement, until it reached the human. If the blood-feeding interaction occurred, the model would change the value of the blood variable of the mosquito, and the human would receive a signal to increase the number of bites variable by 1. In each step, the actions of humans were simulated before those of mosquitoes, and the order within humans and mosquitoes was random. When the step reached 19:00 of each day, an action of recruiting new mosquitoes would follow actions of humans and mosquitoes.

#### Time

The beginning of a simulation was set to be 19:00 on day 1. Night was defined as 19:00 to 05:00, and daytime was defined as 05:00 to 19:00. Time of day was a determinant of the movement rules. Mosquitoes rested during daytime and flew during night. Humans were set to move out of their homes at 07:00 and return home at 20:00. New adult mosquitoes emerged at 19:00 (discussed in Recruitment submodel section).

### Design concepts

#### Basic principles

The theories of sugar feeding, blood feeding, resting, and oviposition of *An. gambiae* mosquitoes are discussed in the Process Overview and Scheduling section. The model simulated all these behaviours and their interactions with the environment within a two-dimensional village setting. The theories and hypotheses were also used in the submodels described below. The simulations were set to represent real-world scenarios and thus could be used to predict the impact of environmental sugar sources and outdoor resting sites on the survival and human biting behaviour of *An. gambiae*.

#### Emergence

The emergent output of most interest were the effects of numbers and spatial distributions of natural resting sites and sugar sources on the survival and human biting rates of *An. gambiae* mosquitoes, which vary in unpredictable ways as the environmental configuration changes.

#### Adaptation

The mosquito agents could make some simple adaptive decisions. As per the sample described in Sequence of Actions in the Process Overview and Scheduling section states, in one step, a female *An. gambiae*, according to it status, could decide to seek a resource or stay static. If the female mosquito was in need of a blood meal, then she would search the surrounding environment and see if there was a human within range. If there was then the female mosquito would move one meter toward the human, and continue to fly toward the human in the following steps until she reached the human. If the human was moving, then the mosquito would target the new location of the human in each step.

#### Objectives

The objectives calculated by the model were the daily abundances of mosquitoes and human biting rates. The adaptation traits of the individuals did not increase their success at meeting the objectives. The objectives were measured by recording the number of mosquitoes and total number of bites every day.

#### Learning

No learning behaviour by individuals was built into the model.

#### Prediction

The mosquito agents could make simple predictions about the location of humans, sugar sources, resting sites, and larval habitat sites from sensory input.

#### Sensing

The mosquitoes could sense humans, sugar sources, resting sites, and larval habitat sites within certain radiuses and move in the direction of the targets.

#### Interaction

The mosquitoes had interactions with humans through blood feeding, with sugar sources for sugar feeding, with resting sites for resting, and with larval habitat sites for oviposition. The blood feeding interaction affected the bite counter of the humans, and the ovipositing interaction affected the egg count of the larval habitat sites.

#### Stochasticity

The outdoor resting sites and sugar sources were assigned randomly for each simulation. Humans moved randomly outside of their houses. Mosquito movement was partly random, but partly directed when a target was detected.

#### Collectives

No intermediate collectives were considered.

#### Observation

Daily abundances of mosquitoes were recorded before and after recruitment each day. Daily number of bites was recorded at every blood-feeding event. Egg count was recorded for each larval habitat site at every oviposition event.

### Initialization

**Figure 2:**
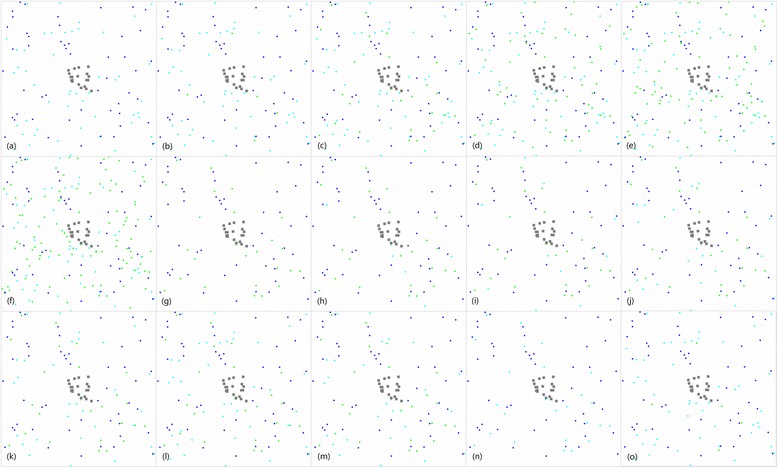
**Landscapes for scenarios of different distributions of sugar sources and outdoor resting sites.** The whole square represents the village, the grey dots in the center represent houses, the green dots represent natural sugar sources, the light blue dots represent outdoor resting sites, and the dark blue dots represent breeding sites. Subfigures **(a)** to **(f)** represents sugar source numbers of 0, 5, 25, 50, 75 and 100, respectively, with 50 randomly located outdoor resting sites. Subfigures **(g)** to **(l)** and **(c)** represent outdoor resting site numbers of 0, 5, 10, 20, 30, 40, respectively, with 25 randomly located sugar sources. Subfigures **(m)** and **(n)** represent random distributions and adjacent distributions of sugar sources with outdoor resting sites. Dots representing sugar sources are hidden behind the outdoor resting sites when they have the same location. Subfigures **(c)** and **(o)** represent random distribution and adjacent distribution of sugar sources with houses. Dots representing sugar sources are hidden behind houses when they have the same location.

Different scenarios were simulated to test the impact of sugar sources and outdoor resting sites (Figure 2), and each scenario was repeated 50 times. A total of 1,000 male and 1,000 female adult *An. gambiae* mosquitoes were released at the beginning of each simulation. The age, sugar, blood, gravid status, and location variables of each mosquito were randomly assigned and could differ between each repetition and simulation.

Location and a home were randomly assigned for each human at the beginning of simulation and could differ between each repetition and simulation. Fifty randomly located larval habitat sites were used through all simulation scenarios and repetitions.

Scenarios were initiated with different numbers of outdoor resting sites and sugar sources.

#### Sugar sources

To estimate the impact of different densities of sugar sources, a fixed number of 50 outdoor resting sites were used, and the locations were randomly selected and constant throughout all the simulations and repetitions of different densities of sugar sources. Sugar sources were simulated at six scenarios 0, 5, 25, 50, 75 and 100. The locations of sugar sources were randomly selected for each scenario and kept constant through repetitions.

#### Outdoor resting sites

To estimate the impact of different densities of outdoor resting sites, a fixed number of 25 sugar sources were used, and 0, 5, 10, 20, 30, 40 and 50 outdoor resting sites were tested as six scenarios. The randomness and repetitions of these simulations were similar to the sugar source simulations described above.

To estimate the impact of closeness between outdoor resting sites and sugar sources, 25 sugar sources and 25 outdoor resting sites at the same or random locations were simulated.

To estimate the impact of closeness between sugar sources and houses, one sugar source by each of the 20 houses with the other 5 randomly located and 25 randomly located sugar sources were simulated, and 50 outdoor resting sites were randomly located in both simulations.

### Input data

The model does not use external input data to represent time-varying processes.

### Submodels

#### Resource-seeking submodel

When an *An. gambiae* decided to sugar feed, blood feed, oviposit or rest, it would begin a resource-seeking procedure for the targeted object. The attractive distances of sugar sources, humans, larval habitat sites and resting sites were set to be 5 metres, 40 metres, 5 metres and 5 metres, respectively, according to the preliminary field study results (Günter C. Müller, unpublished data). *Anopheles gambiae* would search its surrounding environment within the radius of the attractive distance of its target. If there was no target in the circle, it would move randomly to one of the eight adjacent one-meter grid cells. If there was one target in the circle, it would move one grid cell towards the target and continue to move towards it in the following steps. If there were more than one target in the circle, it would first select one target randomly, and then move toward the target. Random selection was believed to be more realistic than selecting the nearest one because in front of many targets, mosquitoes always have a preference. For example, humans with higher CO_2_ output are more attractive [39]. The randomly selected target was assumed as the preferred target. Also, successful sugar feeding, blood feeding, ovipositing and resting were always assumed if *An. gambiae* arrived at the same location of the target.

#### Recruitment submodel

New adult *An. gambiae* would be recruited and emerge at 19:00 every day. Although *An. gambiae* mosquitoes actually emerge throughout the night instead of at one time point [40], they normally rest during the first night [41], so the assumption didn’t affect the magnitude of results. However, the mosquito abundance would change sharply instead of smoothly because of this simplification. As the development time of eggs leading to adult emergence is about 12 days [40,42], for each of the first 12 days, there were no data on the oviposited egg counts 12 days before (simulation not started), so the number of recruited mosquitoes was assumed to equal the number of deaths of the prior day to achieve a steady state equilibrium population. From day 13, the number of recruited mosquitoes was a function of the total number of eggs oviposited 12 days before. Density-dependent development of the aquatic stages was accounted for in the function by setting a maximum egg capacity of each breeding site as 400. This number was assumed because further details of type, area, predator, etc. of each breeding site was not included in this model. When the average number of eggs of the ovipositing day (12 days before the recruiting day), one day before, and one day after the ovipositing day exceeded the maximum egg capacity (400), then only 400 eggs would be counted as the effective egg number. When the average number of eggs was smaller than the maximum egg capacity, then the number of eggs of the ovipositing day would be used. Accounting for environmental factors such as drying of temporary breeding sites and the competition of the different aquatic stages of *An. gambiae*, 5% of the eggs were assumed to develop to adult *An. gambiae* [40]. Equal numbers of new males and females were assumed. For example, if the average number of total oviposited eggs at one site at day 3, day 4 and day 5 exceeded 400, then only 400 would be counted as the number of effective eggs oviposited at day 4, and the number of recruits of day 16 (12 days after day 4) would be 5% of 400 which is 20; if average number of total oviposited eggs at day 3, day 4 and day 5 was smaller than 400, then the number of recruits of day 16 would be 5% of the number of oviposited eggs at day 4. Half of the recruited mosquitoes would be males, and the other half would be females.

A summary of input parameters was provided in Table 1.

**Table 1:** **Parameter input used in the model**

**Input/parameter**	**Value**	**Reference**
Human moving outdoors	07:00 to 20:00	Assumption
Active time of *An. gambiae*	19:00 to 05:00	Assumption
Life span of male *An. gambiae*	7 days	[34]
Life span of female *An. gambiae*	21 days	[34]
Threshold of blood-seeking female switching to sugar-seeking	5 hours	[35] and assumption
Minimum number of sugar meal of female *An. gambiae* per night	2	[36]
Minimum number of sugar/blood meal of male *An. gambiae* per night	1	[36]
Days needed to develop eggs after blood-feeding	2 ~ 3 days	[37]
Average size of egg batches	100	[38]
Attractive distance of sugar source	5 m	Unpublished study results
Attractive distance of human	40 m	Unpublished study results
Sensing distance of larval habitat site	5 m	Unpublished study results
Sensing distance of resting site	5 m	Unpublished study results
Days of aquatic cycle	12 days	[40,42]
Egg capacity of breeding site	400	Assumption
Percentage of eggs developing to adults	5%	[40]

### Program

JAVA 7 (Oracle Co., Redwood, CA) and Mason package v17 [43] were used to develop this model.

### Statistical analysis

Daily survival rate was defined as the number of *An. gambiae* mosquitoes at the end of day (before recruits) divided by the number at the beginning of the day (right after recruits). Human biting rate was defined as the total number of bites per day divided by the number of humans. Daily abundance was defined as the number of *An. gambiae mosquitoe*s at the end of each day. Daily survival rates and abundances were calculated separately for males and females.

Because in the first 12 days, the number of deaths of the former day instead of the function of the number of eggs oviposited 12 days before was used to calculate the number of recruits, simulation was not realistic for the first 12 days. Hence only data from day 13 on were used for the analysis. The mean daily survival rate, human biting rate and daily abundance were calculated for each scenario; records from day 13 to day 60 for all 50 repetitions were used for the calculation. For comparison of average daily survival rates, human biting rates and abundances between scenarios, average area under curve (AUC) of the 48 days used was calculated for each repetition of simulation. ANOVA was used for the comparison, and the Tukey post hoc test was used to compare between each density level of sugar sources and outdoor resting sites.

Generalized mixed linear regression model was used to control potential influence of time. Scenario, time (days) and their interaction term was included in the models. Time (days) was used as a repeated variable, and repetition within each scenario was used as subject variable. Either a first-order autoregressive structure, a compound-symmetry structure, or a variance components structure was used as the covariance structure, depending on which structure gave the best fit (smallest AIC). F test was used to examine the significance of each term. Least square means of each outcome variable were compared between scenarios using t tests. A scenario of five sugar sources or outdoor resting sites was used to replace the scenario of 0 sugar sources or outdoor resting sites to improve the model fit.

SAS 9.3 (SAS Institute, Inc., Cary, NC) was used for the analyses.

## Results

**Figure 3:**
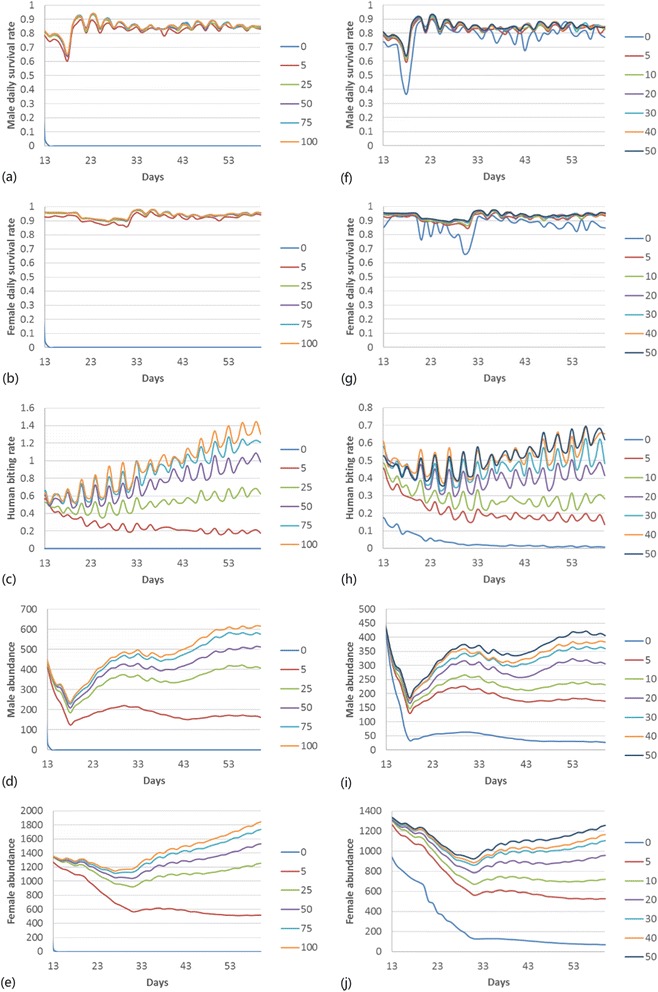
**Daily survival rates, human biting rates, and daily abundances of** ***An. gambiae*** **in different densities of sugar sources and outdoor resting sites.** Subfigures **(a)** to **(e)** represent male daily survival rates, female daily survival rates, human biting rates, male daily abundance, and female daily abundance, respectively, of *An. gambiae* in environments of different sugar source densities. Subfigures **(f)** to **(j)** represent male daily survival rates, female daily survival rates, human biting rates, male daily abundance, and female daily abundance, respectively, of *An. gambiae* in environments of different outdoor resting site densities. Each line represents one outcome in one density scenario.

Table 2 shows the means and standard deviations of daily survival rates, human biting rates and daily abundances of different scenarios. Survival and human biting rates of *An. gambiae* increased with the increase of sugar source density and resting site density, even the densities increased by only 5 from 0, the survival and human biting increased substantially. Daily abundances were higher when sugar sources were randomly distributed from outdoor resting sites, and both daily abundances and human biting rates were higher when sugar sources were randomly distributed from houses. Figure 3 shows the variation of survival and human biting rate along time with different sugar source or resting site densities. The order of magnitude of the five outcomes remains the same through time.

**Table 2:** **Means of daily survival rate, human biting rate and daily abundance in different scenarios**

**Scenarios**		**Male daily survival rate (%)**		**Female daily survival rate (%)**		**Human biting rate**		**Male abundance**		**Female abundance**	
		**Means**	**SD**	**Means**	**SD**	**Means**	**SD**	**Means**	**SD**	**Means**	**SD**
Sugar resource density	0	2.53	3.67	2.53	3.67	0.00	0.00	0.82	7.60	0.82	7.60
	5	82.37	10.24	91.95	8.48	0.26	0.29	187.44	120.76	703.38	411.23
	25	84.31	6.97	93.85	4.47	0.52	0.78	349.47	305.27	1119.95	806.10
	50	84.65	6.78	94.15	3.87	0.75	1.42	408.84	419.02	1265.72	1093.40
	75	84.94	6.34	94.40	3.41	0.88	1.81	454.90	478.49	1372.15	1245.94
	100	85.05	6.37	94.48	3.32	0.94	1.99	477.27	501.11	1427.82	1316.65
Resting site density	0	77.43	18.36	86.73	13.65	0.04	0.07	62.48	81.15	256.59	283.14
	5	82.47	9.83	91.73	7.58	0.21	0.24	197.06	140.56	698.35	431.75
	10	83.14	8.78	92.59	6.87	0.29	0.36	236.19	180.27	822.90	520.47
	20	83.77	8.19	93.31	6.51	0.41	0.59	283.85	222.83	951.47	616.45
	30	84.05	7.60	93.51	5.95	0.47	0.68	316.76	268.01	1038.05	726.16
	40	84.16	7.28	93.66	5.07	0.52	0.77	329.85	275.02	1069.11	728.00
	50	84.31	6.97	93.85	4.47	0.52	0.78	349.47	305.27	1119.95	806.10
Closeness between sugar resources and resting sites	random	83.84	7.81	93.37	6.41	0.45	0.67	295.23	250.10	983.42	684.06
	adjacent	84.03	8.83	92.41	4.56	0.48	0.92	271.74	287.03	738.05	528.93
Closeness between sugar sources and houses	random	84.31	6.97	93.85	4.47	0.52	0.78	349.47	305.27	1119.95	806.10
	adjacent	82.65	9.34	92.31	7.34	0.27	0.31	203.42	129.55	742.89	411.56

ANOVA of average AUCs shows that both the impacts of increasing the sugar source density and resting site density were substantial (Table 3). According to post hoc analysis, daily survival of both male and female *An. gambiae* was significantly higher in environments with sugar source densities of 5, 25, 50, 75 and 100 than density of 0; the daily survival was also significantly higher in environments with sugar source densities of 25, 50, 75 and 100 than in sugar source density of 5. Difference in daily survival was not significant between sugar source densities of 25, 50, 75 and 100. Human biting rate was only significantly different between scenarios of density 0 and density 25 and higher, between density 5 and density 100. For different densities of resting sites, daily survival was only significantly different between density 0 and other density levels, human biting rate was only significantly different between density 0 and density 20 and higher.

**Table 3:** **Comparison of average AUCs of daily survival rate, human biting rate and daily abundance in different scenarios**

**Scenarios**		**Male daily survival rate**	**Female daily survival rate**	**Human biting rate**	**Male abundance**	**Female abundance**
Sugar resource density	0	0.0	0.0	0.0	19.6	19.6
	5	38.2	42.7	11.9	8708.5	32869.0
	25	39.6	44.1	24.4	16355.9	52462.2
	50	39.8	44.2	35.0	19148.1	59313.7
	75	39.9	44.4	41.1	21326.1	64317.8
	100	40.0	44.4	44.3	22379.7	66938.4
	SE	0.3	0.3	7.9	2188.5	5489.0
	F	3023.89	3281.72	4.83	15.73	21.77
	P	<.0001	<.0001	0.0003	<.0001	<.0001
Resting site density	0	31.0	38.2	1.7	2781.8	11814.8
	5	38.6	43.0	9.9	9159.1	32628.5
	10	39.1	43.3	13.5	11007.2	38490.1
	20	39.2	43.6	19.2	13255.8	44537.5
	30	39.5	43.9	21.8	14807.7	48612.5
	40	39.6	44.0	24.2	15424.6	50068.3
	50	39.6	44.1	24.4	16355.9	52462.2
	SE	0.6	0.6	3.5	1364.5	3522.6
	F	25.84	14.50	5.90	11.99	16.15
	P	<0.0001	<.0001	<.0001	<.0001	<.0001
Closeness between sugar resources and resting sites	random	39.2096	43.70895	20.83467	13794.52	46042.25
	adjacent	39.39681	43.41708	22.36233	12642.16	34496.53
	SE	0.327913	0.324676	5.160015	1702.99	3545.201
	F	0.16	0.4	0.04	0.23	5.3
	P	0.6873	0.5265	0.8346	0.6334	0.0234
Closeness between sugar sources and houses	random	39.6	44.1	24.4	16355.9	52462.2
	adjacent	38.6	43.1	12.6	9465.6	34727.9
	SE	0.4	0.4	3.7	1435.6	3684.0
	F	3.59	2.71	5.07	11.52	11.59
	P	0.0611	0.1027	0.0266	0.0010	0.0010

ANOVA of average AUCs also shows that distributions of sugar sources and outdoor resting sites at the same locations or at random locations did not have a significant impact on daily survival rates, human biting rate or male abundance, but female abundance was significantly higher when sugar sources were randomly distributed in the whole village (Table 3). Having sugar sources randomly distributed in the whole village also resulted in higher survival and human biting behaviours than clustering sugar sources around houses, but only the differences of human biting rate, male and female abundance were significant (Table 3).

According to results of generalized mixed linear regression model, scenario and time were both significant factors of outcomes of daily survival rates, human biting rates and daily abundances. The interactions between scenario and time were significant factors of predicting human biting rates and abundances. After controlling the effect of time, scenario was still a significant factor of affecting the outcomes (Table 4).

**Table 4:** **Generalized mixed linear regressions of influence of scenario and time on daily survival rates, human biting rates and daily abundances (F(df);P)**

	**Sugar source density**			**Resting site density**		
	**Scenario**	**Day**	**Scenario × day**	**Scenario**	**Day**	**Scenario × day**
Male survival rate	43.24(4);<0.0001	148.08(47);<0.0001	0.89(188);0.8451	1.75(5);0.1225	227.77(47);<0.0001	0.91(235);0.8365
Female survival rate	22.49(4);<0.0001	46.61(47);<0.0001	0.96(188);0.645	9.86(5);<0.0001	38.37(47);<0.0001	0.79(235);0.9924
Human biting rate	2.85(4);0.0246	28.38(47);<0.0001	1.66(188);<0.0001	4.32(5);0.0008	33.58(47);<0.0001	1.35(235);0.0003
Male abundance	412.61(4);<0.0001	12.03(47);<0.0001	1.19(188);0.0364	4.92(5);0.0002	148.89(47);<0.0001	5.45(235);<0.0001
Female abundance	3.43(4);0.0083	83.6(47);<0.0001	4.09(188);<0.0001	2.86(5);0.0137	210.74(47);<0.0001	2.46(235);<0.0001

After controlling the effect of time (including time and its interaction with scenario in the generalized mixed linear regression model), a number of differences of the outcomes between different none-zero sugar source or outdoor resting site densities, which were not significant in ANOVA results, became significant. In addition, even when the lowest density applied in this analysis was 5 instead of 0, the outcomes in scenarios with higher densities were still significant greater than the lowest density scenarios (Table 5).

**Table 5:** **Comparison of daily survival rates, human biting rates and daily abundances between different sugar source and resting site densities after controlling time (** ***P*** **)**

	**Sugar source density**					**Resting site density**					
		**s5**	**s25**	**s50**	**s75**		**r5**	**r10**	**r20**	**r30**	**r40**
Male daily survival rate	s25	<0.0001				r10	0.3500				
	s50	<0.0001	0.1661			r20	0.1024	0.4830			
	s75	<0.0001	0.0099	0.2271		r30	0.0364	0.2445	0.6432		
	s100	<0.0001	<0.0001	0.0957	0.6448	r40	0.0255	0.1915	0.5444	0.8863	
						r50	0.0149	0.1314	0.4183	0.7290	0.8388
Female daily survival rate	s25	<0.0001				r10	0.0353				
	s50	<0.0001	0.3772			r20	<0.0001	0.0425			
	s75	<0.0001	0.1107	0.4746		r30	<0.0001	0.0058	0.4605		
	s100	<0.0001	0.0679	0.3434	0.8158	r40	<0.0001	0.0017	0.2582	0.6937	
						r50	<0.0001	0.0003	0.1069	0.3802	0.6284
Human biting rate	s25	0.2635				r10	0.1592				
	s50	0.0387	0.3391			r20	0.0217	0.1592			
	s75	0.009	0.1319	0.5801		r30	0.0034	0.0409	0.5214		
	s100	0.0039	0.0743	0.4049	0.7795	r40	0.0005	0.0086	0.2188	0.5552	
						r50	0.0004	0.0078	0.2065	0.5330	0.9733
Male abundance	s25	<0.0001				r10	0.1478				
	s50	<0.0001	0.0003			r20	0.0041	0.1555			
	s75	<0.0001	<0.0001	0.0001		r30	0.0005	0.0435	0.5491		
	s100	<0.0001	<0.0001	<0.0001	0.1424	r40	0.0002	0.0218	0.3825	0.7839	
						r50	<0.0001	0.0094	0.2386	0.5622	0.7601
Female abundance	s25	0.0601				r10	0.3558				
	s50	0.0112	0.5107			r20	0.0606	0.3405			
	s75	0.0025	0.2551	0.631		r30	0.0118	0.1107	0.5209		
	s100	0.0011	0.1647	0.4645	0.8016	r40	0.006	0.068	0.3831	0.8179	
						r50	0.006	0.0277	0.2117	0.5438	0.7063

## Discussion

This study highlights how the environmental sugar sources and outdoor resting sites affect the survival and human biting rate of *An. gambiae.* When the numbers of sugar sources or resting sites were at low levels, small increase in their densities resulted in significant increase of daily survival rates, human biting rates, and daily abundances of *An. gambiae*. Time had a significant impact on the outcomes and interacted with density scenarios significantly. After eliminating the effect of time, the results show that at higher sugar source or resting site densities, increase in their densities still increased the outcomes, although the differences were less significant. Surprisingly, this model suggested that placing sugar sources at each outdoor resting site or at each house did not increase, but rather decreased survival and human biting rate of *An. gambiae*, although the differences were only significant in three of the five outcomes.

As mentioned, further increase of sugar source and outdoor resting site density from higher density levels (sugar source density of 25, resting site density of 10) did not increase the survival and human biting rates of *An. gambiae* significantly without controlling the effect of time. The reasons for this could be the following: first, the amount of increase could depend on the ratio of vector abundance and resource density. Here, only 1,000 males and 1,000 females were simulated at the beginning, so further availability of sugar sources and resting sites might have been too much and did not have an effect in this model. Second, it could be the assumptions that either sugar sources or resting sites can be reused continuously; that is, as long as the vectors could find the resource, they would be able to use it no matter how many *An. gambiae* mosquitoes were using them at the same time. But in reality, sugar sources like nectars are not persistent, and density of *An. gambiae* at certain resting sites is always limited [44-46]. So, this model may have underestimated the effect of further increase of sugar source and outdoor resting site densities, especially when real abundance of *An. gambiae* is high. Thus, field studies are suggested to determine the density increase of sugar sources or outdoor resting sites that can significantly impact the survival and human biting behaviour of *An. gambiae* in real environment. Instead of an increase of sugar source or outdoor resting site densities, it is also possible to test removing sugar sources or outdoor resting sites in field studies. Or, on the other hand, the model assumptions of initial mosquito abundances and the capability of each sugar source and resting site can be adjusted with field study results and provide more accurate predictions.

As shown in the results, a reduction in the number of sugar sources to very low levels is expected to significantly reduce mosquito numbers. This is apparently difficult in resource-rich settings, where removing certain amount of sugar sources can be meaningless; however, in resource-poor settings, further eliminating sugar sources can greatly reduce mosquito abundance, which can be used as a mosquito control strategy in sugar poor areas. Another consideration is that decreased availability of sugar source may increase blood-feeding behaviour of each female [5]. This is consistent with the results, which shows that sugar source density decrease from 25 to 5 reduced mosquito survival significantly but not the human biting rate. Thus, instead of removing all the sugar sources, placing ATSB stations near natural sugar sources or houses, or spraying ATSB solutions on vegetation may have better sugar blocking and vector control results. For resting sites, only reducing the number of outdoor resting sites to 0 had a significant effect on mosquito survival and human biting. In other words, with houses as indoor resting sites, very small number of outdoor resting sites can support mosquito survival very well. Thus in order to achieve good vector control via manipulating resting sites, disabling indoor resting sites should be emphasized.

Although it may appear that providing sugar sources where the vectors rest can make sugar seeking easier and hence improve their survival, the results indicated that it may not be the case. The explanation is that *An. gambiae* may need sugar for energy before blood feeding or ovipositing, or for flight for other targets. Thus, sugar sources distributed over the whole village can provide a better supporting environment. This result also agrees with some of the preliminary field study observations that mosquitoes in Mali do not sugar feed near their resting sites, and there is no overlap in mosquitoes going to either resource (unpublished data).

Other IVM methods such as LLINs have not been considered in the model, so the human biting rates can be overestimated. Also, field studies providing more information can help to better adjust the model assumptions/rules, for example, the model can be improved by including the features of variation in sugar-source quality and different sugar-feeding patterns, inadequate blood meals and its effect on fecundity. With the large scale and high temporal resolution, the model can be slowed down and it will take longer to complete the simulation than the other simplified models.

While other models use parameters such as daily mortality rate [47,48], which always varies in different environment conditions such as different sugar source availabilities [7], this model only uses the basic characteristics of *An. gambiae* mosquitoes, such as their average life span, and determines the daily mortality rates by the model itself in the given scenarios, by adding the numbers of mosquito deaths due to different reasons, which is more accurate. For example, mosquitoes not able to find an energy resource for a whole night would die of starvation, mosquitoes that reached the maximum age would die of age, and the model recorded all these deaths to calculate the daily mortality rate. Also, human biting rates vary largely among different environmental conditions and mosquito species, while the human biting rates estimated in this model are consistent with the previously recorded range [49].

In other models with temporal resolutions of one hour or even one day, the individuals like the *An. gambiae* mosquitoes can only have one chance to be presented in a specific state and seek for resources or move a grid cell in every hour/day, which can underestimate their success rate of finding a resource, and devoid the capacity to express the impact of the spatial configuration. The reason for these limitations is that resources are located in the large grid cells, and mosquitoes can only find the resource if it is located in the adjacent eight grid cells. However in this model using a one-second temporal resolution, the mosquitoes can check their states, decide their next move and complete an action in every step/second, which is more akin to real conditions. It allows the mosquitoes many chances to reach food, and can take the distance from the resources into account.

With the individual level simulation, all steps an individual mosquito or human takes can be tracked and all the details such as how many female mosquitoes died from starvation, how many mosquitoes feed on one particular sugar source, and even the route of moving of any mosquito during the whole simulation can be obtained. This capability of this model allows the development of many other hypotheses and their examination.

## Conclusions

According to this model, increases in the number of sugar sources and resting sites in resource poor scenarios significantly promotes the survival of *An. gambiae*, increases their population, and increases the rate of human biting. This suggests methods of removing sugar sources in sugar poor areas and the use of ATSB to target the sugar feeding behaviour. To target the resting behaviour of the vectors, emphasis should be put on indoor resting sites because with the availability of indoor resting sites, very small number of outdoor resting sites can provide good support for the survival and human biting of *An. gambiae*. The results show that when sugar sources and outdoor resting sites are distributed over the whole village, they offer better support for *An. gambiae* than when they are limited to certain areas, even located by each outdoor resting site or house. This observation emphasizes the importance of spatial configuration of resources in vector control. To target sugar sources or resting sites for vector control, field studies with real configurations of the environment and mosquito abundance are needed. Such studies will enable the determination of the level of density decrease in sugar sources or outdoor resting sites that can have a significant effect on mosquito population.

## Abbreviations

ABM/IBM: Agent/individual-based model
AIC: Akaike information criterion
ATSB: Attractive toxic sugar bait
AUC: Area under curve
IRS: Indoor residual spraying
ITN: Insecticide-treated net
IVM: Integrated vector management
LSM: Larval source management
ODD: Overview design concepts, and details
